# SARS-CoV-2-assoziierte Todesfälle von erwachsenen Personen bis 50 Jahre: Eine retrospektive Auswertung der COVID-19-assoziierten Todesfälle im Jahr 2020 im Institut für Rechtsmedizin Hamburg, Deutschland

**DOI:** 10.1007/s00194-021-00483-8

**Published:** 2021-04-13

**Authors:** L. Lohner, D. Fröb, C. Edler, A. S. Schröder, E. Dietz, B. Ondruschka

**Affiliations:** Institut für Rechtsmedizin Hamburg, Universitätsklinikum Hamburg-Eppendorf, Butenfeld 34, 22529 Hamburg, Deutschland

**Keywords:** Todesfälle bei jungen Personen, SARS-CoV‑2-Obduktionen, SARS-CoV-2-Infektionen, Komorbiditäten, Adipositas, Deaths in young persons, SARS-CoV-2 autopsies, SARS-CoV‑2-infections, Comorbidities, Obesity

## Abstract

**Einleitung:**

In der internationalen Literatur finden sich mehrere Auswertungen zu SARS-CoV-2-assoziierten Todesfällen bei Personen in hohem Lebensalter. Ziel dieser Arbeit war die Evaluation SARS-CoV-2-assoziierter Todesfälle von Personen jüngeren oder mittleren Lebensalters (bis 50 Jahre) im Institut für Rechtsmedizin in Hamburg mit Darstellung der Häufigkeit, der Komorbiditäten und der Krankheitsverläufe.

**Material und Methoden:**

Durch das Institut für Rechtsmedizin in Hamburg wurden im Jahr 2020 insgesamt 735 SARS-CoV-2-assoziierte Fälle von Verstorbenen mit Hamburger Meldeadresse anhand verschiedener Untersuchungsmethoden evaluiert. Die Auswahl und Durchführung der jeweiligen Untersuchungsmethoden erfolgten je nach Zustimmung durch die nächsten Angehörigen. Darüber hinaus wurden weitere Sektionen von Verstorbenen mit externer Meldeadresse und positivem SARS-CoV-2-Nachweis durchgeführt.

**Ergebnisse und Schlussfolgerungen:**

Neun der 735 Verstorbenen mit Hamburger Meldeadresse und 3 der untersuchten Todesfälle mit externer Meldeadresse (*n* = 12; 7 Männer und 5 Frauen) waren ≤50 Jahre alt, mit einem Altersdurchschnitt von 39,8 Jahren. Im Wesentlichen bestanden kardiovaskuläre, neurologische und maligne Erkrankungen sowie eine Adipositas. Bei 2 Todesfällen wurde SARS-CoV‑2 erstmalig postmortal nachgewiesen; bei diesen beiden fand sich eine virusunabhängige Todesursache. Sieben der Personen verstarben an einer COVID-19-Pneumonie, 3 Personen an den Folgen der notwendigen intensivmedizinischen Behandlung.

Mehrere Studien konnten insbesondere bei jüngeren Patienten einen Zusammenhang zwischen Übergewichtigkeit und schweren SARS-CoV-2-bedingten Krankheitsverläufen zeigen, was sich auch im hiesigen rechtsmedizinischen Untersuchungskollektiv bestätigte.

## Abstract (en)

**Introduction:**

Several evaluations of deaths in persons of advanced age associated with SARS-CoV‑2 can be found in the international literature. The aim of this work was the evaluation of deaths associated with SARS-CoV‑2 of persons of younger or middle age (up to 50 years) at the Institute of Legal Medicine in Hamburg, Germany, with presentation of frequency, comorbidities and disease courses.

**Material and methods:**

A total of 735 SARS-CoV-2-associated cases of decedents with registered addresses in Hamburg were evaluated in 2020 at the Institute of Legal Medicine in Hamburg, Germany, using various examination methods. The selection and performance of the respective methods was based on the consent given by the relatives. In addition, more autopsies of decedents with a registered address outside Hamburg and positive SARS-CoV‑2 detection were performed.

**Results and conclusion:**

Of the 735 decedents 9 with a registered Hamburg address and 3 of the deaths studied with an external registered address (*n* = 12; 7 men and 5 women) were aged 50 years or younger, with an average age of 39.8 years. Essentially, there were cardiovascular, neurological, and malignant pre-existing diseases, as well as obesity. The SARS-CoV‑2 was detected post-mortem for the first time in two cases; these were found to have a virus-independent cause of death. Of the individuals 7 died from COVID-19 pneumonia, 3 individuals from the consequences of the necessary intensive medical treatment.

Several studies have demonstrated an association between obesity and severe SARS-CoV-2-related disease progression, particularly in younger patients and this was confirmed in the legal medicine study population.

## Einleitung

Am 11.03.2020 erklärte die World Health Organization (WHO) den Ausbruch des „severe acute respiratory syndrome coronavirus 2“ (SARS-CoV-2) zu einer weltweiten Pandemie [1]. Seitdem existieren in der Literatur mehrere Untersuchungen und Fallberichte zu SARS-CoV-2-Infektionen und assoziierten Todesfällen [2–23]. Die unterschiedlichen Autoren betonen wiederholt die Wichtigkeit der Sektionen, um das neuartige Coronavirus, seine Wirkungsweise und seine Manifestationen an den Organen zu verstehen und um ggf. neue Therapieansätze für infizierte Personen zu finden. Obduktionen von *mit* und *an* „coronavirus disease 2019“ (COVID-19) Verstorbenen sind nicht nur von wissenschaftlicher Relevanz, sondern auch für die korrekte statistische Erfassung von Sterbefällen von erheblicher Bedeutung, für gesundheitspolitische Entscheidungen und eine sachgemäße Todesursachenstatistik [4]. Gemäß größerer, internationaler Studien waren Personen, die *mit* oder *an* einer SARS-CoV-2-Infektion verstarben, zwischen 52 und 96 Jahre alt, mit einer leichten Dominanz des männlichen Geschlechts [4, 10, 16, 22, 24]. Zu SARS-CoV-2-assoziierten Todesfällen von Verstorbenen unter 50 Jahren liegen Einzelfallberichte, aber keine systematischen Übersichten vor. Barton et al. berichteten den Fall eines 42-jährigen verstorbenen Mannes mit SARS-CoV-2-Nachweis und einer myotonen Muskeldystrophie [2]. Xu et al. beschrieben einen SARS-CoV-2-assoziierten Todesfall eines 50-jährigen Mannes, ohne Angaben zu Komorbiditäten [20]. Größere Studien, die sich explizit auf jüngere Verstorbene mit SARS-CoV-2-Nachweis beziehen, existieren aktuell in der deutsch- und englischsprachigen wissenschaftlichen Literatur unseres Wissens nach nicht. Ein hohes Lebensalter wurde als ein wichtiger Einflussfaktor für die Entwicklung schwerer Pneumonien, die Hospitalisierungsrate und Mortalität bei SARS-CoV-2-Nachweis berichtet [9, 18, 22]. Frühere Studien an Makaken, die mit SARS-CoV infiziert wurden, ergaben, dass ältere Tiere eine stärkere Wirtsreaktion auf die Virusinfektion zeigten als jüngere [25]. Neben einem erhöhten Lebensalter existieren weitere Risikofaktoren, die mit COVID-19-assoziierten Todesfällen im Zusammenhang stehen. Hierbei handelt es sich v. a. um Komorbiditäten wie arterielle Hypertonie, Diabetes mellitus, koronare Herzerkrankung (KHK), chronisch obstruktive Lungenerkrankung (COPD) sowie chronische Nieren- und Lebererkrankungen [3, 14, 22, 26, 27]. Andere Untersuchungen haben Übergewicht und Adipositas, insbesondere bei Patienten unter 60 bzw. 45 Jahren, als erhöhtes Risiko für eine SARS-CoV-2-Infektion und die damit verbundene (intensivmedizinische) Krankenhausaufnahme beschrieben, ohne explizit auf Sterbefälle dieser Patientengruppe einzugehen. Eine höhere Mortalität bei übergewichtigen bzw. adipösen Patienten im Vergleich zu Normalgewichtigen konnte hingegen nicht festgestellt werden [12, 18, 19].

Ziel dieser vorliegenden Auswertung war die Darstellung der Häufigkeit der SARS-CoV-2-assoziierten Todesfälle bei erwachsenen Personen in jüngerem Lebensalter (≤50 Jahre) unter Beleuchtung der Komorbiditäten, der Krankheitsverläufe und möglicher Spezifika aus dem Hamburger Sektionsgut.

## Untersuchungen im Institut für Rechtsmedizin in Hamburg

Am 16.03.2020 wurde der erste SARS-CoV-2-assoziierte Todesfall in Hamburg obduziert [7, 16, 17]. Ab diesem Zeitpunkt wurden im Jahr 2020 alle Hamburger SARS-CoV-2-assoziierten Todesfälle im Auftrag der Behörde für Arbeit, Gesundheit, Soziales, Familie und Integration (Sozialbehörde) der Freien und Hansestadt Hamburg durch das Institut für Rechtsmedizin in Hamburg anhand der ärztlichen Unterlagen (Arztbriefe, Laborbefunde), der postmortalen Computertomographie (PMCT), der ultraschallgestützten minimal-invasiven Autopsie (MIA) und der konventionellen Sektion mit histopathologischen Folgeaufträgen evaluiert. Grundlage für die Untersuchungen und Obduktionen waren ganz überwiegend die jeweilige Beauftragung oder Anordnung der zuständigen Hamburger Gesundheitsämter nach § 25 (4) des Gesetzes zur Verhütung und Bekämpfung von Infektionskrankheiten beim Menschen (IfSG) sowie die Zustimmung der nächsten Angehörigen zu den postmortalen Untersuchungen. Die Auswahl und Durchführung der jeweiligen Untersuchungsmethoden erfolgten je nach Zustimmung der Angehörigen – teilweise wurden mehrere Untersuchungsmethoden pro Fall kombiniert. Darüber hinaus wurden einige SARS-CoV-2-Sterbefälle auch als gerichtliche Sektionen im Auftrag von Staatsanwaltschaften durchgeführt. Nach den Untersuchungsergebnissen wurden die Sterbefälle jeweils in eine der 3 Kategorien eingestuft und an das zuständige Gesundheitsamt gemeldet (Tab. 1).

**Table 1:** 

Kategorie	Erläuterung
*COVID-19-Sterbefall*	COVID-19 todesursächlich
*SARS-CoV-2-Nachweis ohne todesursächliche Bedeutung*	Todesursache, die nicht durch das SARS-CoV‑2 bedingt ist
*Unklar*	Die Todesursache verbleibt unklar. Es gibt keinen substanziierbaren COVID-19-Verdacht

## Material und Methoden

Vom 16.03.2020 an wurden bis über den Jahreswechsel alle aus dem Kalenderjahr 2020 bekannt gewordenen Sterbefälle mit Hamburger Meldeadresse und positivem prä- und/oder postmortalen SARS-CoV-2-Nachweis untersucht. Bis zum April 2020 erfolgten stichprobenartige PCR-Tests auf SARS-CoV‑2 der in das Institut für Rechtsmedizin eingelieferten Verstorbenen. Ab April 2020 wurden alle Verstorbenen routinemäßig auf SARS-CoV‑2 getestet (auch die Verstorbenen, die zu Lebzeiten bereits positiv getestet wurden).

Insgesamt wurden 735 SARS-CoV-2-assoziierte Todesfälle mit Hamburger Meldeadresse und einem Altersmedian von 83 Jahren im Institut für Rechtsmedizin in Hamburg evaluiert. 262 Todesfälle wurden lediglich anhand ärztlicher Unterlagen, 411 anhand postmortaler Computertomographie, 41 anhand ultraschallgestützter minimal-invasiver Autopsie und 283 anhand der konventionellen Sektion evaluiert. In allen Todesfällen wurden, wenn möglich und sofern vorhanden, Krankenunterlagen hinzugezogen und ausgewertet. Im Rahmen der minimal-invasiven Untersuchungen und der konventionellen Sektionen wurden Gewebestanzen bzw. Gewebeproben für nachfolgende histopathologische Untersuchungen asserviert und aufgearbeitet; über die Ergebnisse wird gesondert berichtet. Eine Beurteilung/Klassifikation aller untersuchten Sterbefälle erfolgte anhand eines von Edler et al. [4] modifizierten Schemas (Tab. 1). 344 der 735 (46,8 %) Verstorbenen waren weiblich und 391 (53,2 %) männlich. Alle Sterbefälle mit einem Alter ≤50 Jahre (*n* = 9) wurden nach folgenden Kriterien aufgearbeitet: Sterbedatum, Alter, Geschlecht, Body-Mass-Index (BMI), Sterbeort (kategorial), Todesursache, Krankheitsverlauf, erster SARS-CoV-2-Nachweis, Vorerkrankungen (nach Organsystemen), Überlebenszeit (nach Infektionsnachweis). Darüber hinaus sind 3 weitere SARS-CoV-2-assoziierte Sterbefälle von Personen in einem Alter ≤50 Jahre mit einer externen Meldeadresse und Sterbeort Hamburg aus dem Hamburger Sektionsgut im Jahr 2020 bekannt. Diese wurden als gerichtliche bzw. private Sektionen in Auftrag gegeben. Eine detaillierte Übersicht der 12 Todesfälle findet sich in Tab. 2. Weiterhin ist in Hamburg aus 2020 der Sterbefall eines 7‑jährigen Kindes mit externer Meldeadresse bekannt. Da es sich um ein Kind handelt, wurde dieser Fall bewusst nicht in die vorliegende Auswertung inkludiert. Aufgrund fehlender Zustimmung durch die Angehörigen konnte keine Obduktion erfolgen.

**Table 2:** 

Fall	Meldeadresse	Alter in Jahren/Geschlecht	BMI (kg/m^2^)	RMU	PMCT	Todesursache	Erster positiver SARS-CoV-2-Nachweis	PMI (Tage)	Aufenthaltsdauer in der Klinik	Sterbeort	Autoptischer Nachweis von Lungenembolie/Thrombose	Vorerkrankungen, Sektionsbefunde
*1*	Hamburg	36, weiblich	67,9 (Adipositas Grad III)	Obduktion	–	Dekompensierte Herzinsuffizienz bei COPD	Nachweis postmortal	4,6	–	Häuslichkeit	Nein	Herzinsuffizienz
												Lipomatosis cordis
												Koronarsklerose
												COPD mit Heimsauerstoffbeatmung
												Fettleber
												Hypophysenadenom
*2*	Hamburg	31, männlich	20,6 (Normalgewicht)	Obduktion	+	COVID-19-Pneumonie	5 Tage prämortal (klinisch)	1	PS (7 Tage) ITS (4 Tage)	ITS	Nein	Primär mediastinaler metastasierter Keimzelltumor mit Z. n. Teilentfernung Lunge
												Z. n. TVT
*3*	Hamburg	48, männlich	42,5 (Adipositas Grad III)	Obduktion	+	Sepsis und multiple Hirninfarkte bei COVID-19-Pneumonie	22 Tage prämortal (klinisch)	7	NS (35 Tage) und ITS (16 Tage)	ITS	TVT Periphere LAE	Akute myeloische Leukämie
												Herzhypertrophie
												Lipomatosis cordis
												Arterio- und Koronarsklerose
												Fettleber
*4*	Hamburg	29, weiblich	40,9 (Adipositas Grad III)	Obduktion	+	Kombiniert infektiöses und hypoxisches MOV bei COVID-19-Pneumonie	16 Tage prämortal (klinisch)	2,4	ITS (13 Tage)	ITS	Nein	Pulmonale Hypertonie
												Fettleber mit Zwerchfellhochstand
*5*	Hamburg	47, männlich	–^a^	Obduktion	–	Respiratorische Insuffizienz bei COVID-19-Pneumonie und Vollatelektase rechts, begleitender Pneumothorax bei bullösem Lungenemphysem	2 Tage prämortal (klinisch)	5,8	ITS (2 Tage)	ITS	Nein	Multiple Sklerose
												Anämie
												Herzinsuffizienz
												Z. n. Herzinfarkt
												Arterio- und Koronarsklerose
												pAVK, Z. n. US-Amputationen
												Z. n. Hemikolektomie und Stomaanlage
												Lungenemphysem
*6*	Hamburg	48, weiblich	27,1 (Präadipositas)	Krankenunterlagen	–	Intrazerebrale Blutung unter ECMO bei ARDS durch COVID-19-Pneumonie	28 Tage prämortal (klinisch)	–	ITS (27 Tage)	ITS	–	Chronisch lymphatische Leukämie
												Ausgeprägtes Haut- und Mediastinalemphysem (am ehesten iatrogen)
*7*	Hamburg	46, männlich	28,1 (Präadipositas)	Obduktion	+	COVID-19-Pneumonie	5 Tage prämortal	7,7	–	Häuslichkeit	Thrombosen des Plexus prostaticus	Linksherzhypertrophie
												Arterielle Hypertonie
												Nierenarterienabgangsstenose, Schrumpfniere
*8*	Hamburg	49, weiblich	21,8 (Normalgewicht)	Obduktion	–	Septisches MOV bei COVID-19-Pneumonie	28 Tage prämortal (klinisch)	4,4	ITS (23 Tage)	ITS	TVT beidseits Ältere LAE	Arteriosklerose
												Arterielle Hypertonie
												Lipomatosis cordis
												COPD
												Chronische Pankreatitis
												Äthyltoxische Leberzirrhose
												Z. n. oberer GI-Blutung bei Magenulkus
*9*	Hamburg	50, männlich	32,2 (Adipositas Grad I)	Obduktion	–	Hirnmassenblutung unter ECMO bei COVID-19-Pneumonie	34 Tage prämortal (klinisch)	1	ITS (20 Tage)	ITS	TVT Thrombosen des Plexus prostaticus Zentrale LAE	Herzhypertrophie
												Sigmadivertikulose
*10*	Extern	21, weiblich	26,2 (Präadipositas)	Obduktion	+	COVID-19-Pneumonie	33 Tage prämortal (klinisch)	4,8	NS (12 Tage) ITS (27 Tage)	ITS	Nein	Akute lymphatische Leukämie (B-ALL)
												Z. n. LAE
*11*	Extern	22, männlich	19,7 (Normalgewicht)	Obduktion	+	Hyperglykämie mit oberer GI-Blutung	Nachweis postmortal	2,9	–	Häuslichkeit	Nein	Diabetes mellitus Typ 1
												Kokainabhängigkeit
*12*	Extern	50, männlich	25,8 (Präadipositas)	Obduktion	–	COVID-19-Pneumonie	29 Tage prämortal (klinisch)	0,5	NS (42 Tage) ITS (24 Tage)	ITS	Periphere LAE Thrombosen des Plexus prostaticus	B‑Zell-Lymphom
												Meningeosis lymphomatosa
												Z. n. Stammzelltransplantation
												Arteriosklerose
												Herzinsuffizienz
												Pankreasfibrose
												Chronische Gastritis
												Polyneuropathie

*PMCT* postmortale Computertomographie, *RMU* rechtsmedizinische Untersuchung, *PMI* postmortales Intervall, *COPD* chronisch obstruktive Lungenerkrankung, *ARDS* „acute respiratory distress syndrome“, *ECMO* extrakorporale Membranoxygenierung, *BMI* Body-Mass-Index, *pAVK* periphere arterielle Verschlusskrankheit, *COVID-19* „coronavirus disease 2019“, *MOV* Multiorganversagen, *SARS-CoV‑2* „severe acute respiratory syndrome coronavirus 2“, *US* Unterschenkel, *Z.* *n.* Zustand nach, *GI-Blutung* gastrointestinale Blutung, *TVT* tiefe Beinvenenthrombose, *LAE* Lungenarterienembolie, *ITS* Intensivstation, *NS* Normalstation, *PS* Palliativstation

^a^Aufgrund der Amputation beider Beine ab Höhe der mittleren Oberschenkel konnte kein BMI ermittelt werden

## Ergebnisse

### Demografie

Unter den 735 dokumentierten SARS-CoV-2-assoziierten Todesfällen mit Hamburger Meldeadresse und den untersuchten Sterbefällen mit Nicht-Hamburger Meldeadresse waren insgesamt 12 Verstorbene ≤50 Jahre, mit einem durchschnittlichen Alter von 39,8 Jahren (Range: 21 bis 50 Jahre, Median: 46,5 Jahre). Das Verhältnis Männer zu Frauen betrug 7:5. Der durchschnittliche Body-Mass-Index (BMI) der 12 Verstorbenen lag bei 32,1 kg/m^2^; drei der Verstorbenen zeigten eine morbide Adipositas (BMI >40 kg/m^2^ nach WHO), einer eine Adipositas Grad I. Drei der Verstorbenen waren normalgewichtig und 4 präadipös. Bei einem Verstorbenen konnte postmortal kein BMI bestimmt werden, da zu Lebzeiten die Beine ab mittlerer Höhe der Oberschenkel amputiert wurden.

### Rechtsmedizinische Untersuchung und Todesursachen

In 11 Fällen wurde eine rechtsmedizinische Sektion, inklusive Asservierung von Gewebeproben zur mikroskopischen Untersuchung, durchgeführt; in 6 Fällen erfolgte ergänzend eine postmortale Computertomographie. Ein Fall wurde, bei fehlender Zustimmung zur Obduktion durch die Angehörigen, anhand der umfangreichen Krankenunterlagen nach intensivmedizinischer Behandlung evaluiert.

Todesursachen: In 7 Fällen war die autoptisch festgestellte Todesursache eine COVID-19-Pneumonie. Bei 2 weiteren Fällen war eine medizinische Komplikation bei intensivmedizinischem Behandlungsbedarf einer COVID-19-Pneumonie mittodesursächlich (intrazerebrale Blutung bei extrakorporaler Membranoxygenierung (ECMO), Blutung bei Anlage eines Tracheostoma). Fall 6 (Tab. 1) wurde lediglich anhand der ausführlichen ärztlichen Krankenunterlagen ausgewertet. Klinisch verstarb die Frau an einer in der klinischen Computertomographie nachgewiesenen intrazerebralen Blutung nach ECMO-Anlage bei schwerem „acute respiratory distress syndrome“ (ARDS). Zwei der 11 obduzierten Todesfälle zeigten mit Sektionsbefunden einer dekompensierten Herzinsuffizienz bei chronisch obstruktiver Lungenerkrankung ohne Nachweis eines diffusen Alveolarschadens oder einer Pneumonie und einer Hyperglykämie mit oberer gastrointestinaler Blutung^1^ eine SARS-CoV-2-unabhängige Todesursache. In diesen beiden Fällen war der Virusnachweis erst postmortal erfolgt; typische Symptome der Viruserkrankung hatten anamnestisch zu Lebzeiten nicht vorgelegen.

Insgesamt konnten 10 der 12 Todesfälle als COVID-19-Todesfälle kategorisiert werden. In 5 der 10 COVID-19-Todesfälle wurden autoptisch thrombembolische Ereignisse nachgewiesen (tiefe Beinvenenthrombosen 3/10 (30 %), Thrombosen des Plexus prostaticus 3/6 (50,0 %), periphere bzw. zentrale Lungenarterienembolien 4/10 (40,0 %)).

### Sterbeorte und Zeitraum zwischen erstem positiven SARS-CoV-2-Nachweis und Versterben

Sterbeorte waren in 3 Fällen die Wohnanschrift der verstorbenen Personen und in 9 Fällen die Intensivstation. Bei 2 der in der Häuslichkeit verstorbenen Personen war eine SARS-CoV-2-Infektion zu Lebzeiten nicht bekannt. Diese wurden erst im Institut für Rechtsmedizin durch routinemäßig durchgeführte postmortale nasopharyngeale Abstriche bei Leicheneinlieferung mittels PCR-Tests positiv auf SARS-CoV‑2 getestet. Die durchschnittliche Aufenthaltsdauer der 9 hospitalisierten Personen in der Klinik betrug insgesamt 27,3 Tage (Range: 2 bis 66 Tage), wobei die Aufenthaltsdauer auf der Intensivstation im Durchschnitt 17,3 Tage betrug (Range: 2 bis 27 Tage). Die erste positive Testung auf SARS-CoV‑2 fand im Durchschnitt 20,2 Tage vor dem Versterben statt (Range: 2 bis 34 Tage prämortal).

### Vorerkrankungen/Komorbiditäten

**Figure 1:**
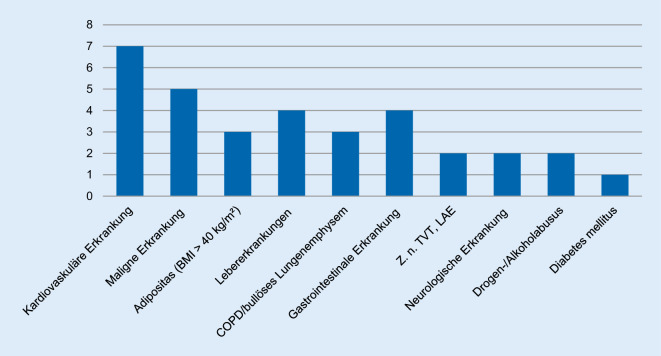

Alle der 12 Verstorbenen waren vorerkrankt. Bei 6 Verstorbenen lagen weniger als 3, bei 5 Verstorbenen ≥5 und bei einem Verstorbenen 3 Komorbiditäten vor (Tab. 2; Abb. 1). In 7 Fällen bestanden kardiovaskuläre Vorerkrankungen (Herzinsuffizienz, Arteriosklerose, Koronarsklerose, periphere arterielle Verschlusskrankheit (pAVK), Herzhypertrophie, Lipomatosis cordis, arterielle Hypertonie). Bei 3 der Verstorbenen lag zudem eine morbide Adipositas vor. Keine der Personen war kachektisch. An weiteren Vorerkrankungen waren eine chronisch obstruktive Lungenerkrankung (COPD), eine Cushing-Krankheit (Hypophysenadenom), eine pulmonale Hypertonie, eine Fettleber/Leberzirrhose, ein Diabetes mellitus Typ 1, Zustand nach tiefen Beinvenenthrombosen (TVT) und Lungenarterienembolie (LAE), eine chronische Gastritis, Magenulkus mit gastrointestinaler Blutung, eine chronische Pankreatitis und eine multiple Sklerose (MS) bekannt. In 5 Fällen bestanden darüber hinaus maligne Vorerkrankungen mit Schwächung des Immunsystems (Keimzelltumor, chronisch lymphatische Leukämie (CML), akute myeloische Leukämie (AML), akute lymphatische Leukämie (ALL), B‑Zell-Lymphom).

Weitere Falldetails werden in Tab. 2 aufgelistet.

## Diskussion

In dem dargestellten Untersuchungskollektiv der SARS-CoV-2-assoziierten Sterbefälle, die in der Hamburger Rechtsmedizin untersucht wurden, fanden sich insgesamt 12 Todesfälle von erwachsenen Personen im Alter bis 50 Jahre. Alle diese 12 Verstorbenen litten zu Lebzeiten an schweren relevanten kardiovaskulären, malignen oder anderen Vorerkrankungen. Teilweise waren diese bekannt; in anderen Fällen konnten autoptisch weitere Erkrankungen festgestellt werden. Bei 8 Verstorbenen lag eine Präadipositas oder eine Adipositas vor. Da das Körpergewicht postmortal im Institut für Rechtsmedizin gemessen wurde, ist hierbei an eine mögliche Gewichtszunahme durch zuvor stattgehabte intensivmedizinische Maßnahmen (Anasarka) zu denken. Angaben zum Körpergewicht bei Krankenhausaufnahme lagen jeweils nicht vor. Zehn dieser 12 Todesfälle wurden als COVID-19-Todesfälle kategorisiert. Die Personen starben an den Folgen einer COVID-19-Pneumonie bzw. an den Komplikationen im Rahmen der notwendigen intensivmedizinischen Behandlungen. In der Hälfte der COVID-19-Sterbefälle (5/10) wurden autoptisch Thrombosen bzw. Lungenarterienembolien festgestellt. Edler et al. berichteten in 40 % thrombembolische Ereignisse in ihrem Sterbefallkollektiv [4]. In allen Fällen lagen schwere Erkrankungsverläufe mit teilweise langen Krankenhausaufenthalten vor. Bei 2 Personen war die Infektion mit SARS-CoV‑2 hingegen nicht todesursächlich; bei diesen wurde eine SARS-CoV‑2 unabhängige Todesursache festgestellt. Der Virusnachweis erfolgte in diesen beiden Fällen erst postmortal bei einem verdachtsunabhängigen Screening im Institut für Rechtsmedizin. Der durchschnittliche Klinikaufenthalt der hospitalisierten Personen bis 50 Jahre von dem ersten positiven SARS-CoV-2-Nachweis bis zum Versterben betrug 27,3 Tage, wobei die mittlere Aufenthaltsdauer auf der Intensivstation 17,3 Tage betrug. Zhou et al. beschreiben mit 18,5 Tagen auf der Normal- bzw. Intensivstation einen vergleichbaren Mittelwert in ihrer Kohorte, wobei es sich hier um 54 Verstorbene in einem medianen Alter von 69 Jahren handelte. 39 dieser Patienten wurden auf der Intensivstation mit einer mittleren Aufenthaltsdauer von 4 bis 12 Tagen behandelt [22]. Auffallend ist die geringe Todeszahl der unter 50-Jährigen in der Hamburger Kohorte, obgleich vom Robert Koch-Institut (RKI) gerade für die „zweite Welle“ der Pandemie in Deutschland auch zahlreiche positive Testergebnisse in dieser Altersgruppe gelistet werden. Am 05.02.2021 meldete das RKI für Deutschland insgesamt 2.264.909 mit SARS-CoV‑2 infizierte Personen, wobei der Anteil der 0- bis 59-Jährigen 73,6 % (1.667.846) ausmacht. Die Altersgruppe der 35- bis 59-Jährigen ist mit 51,2 % am häufigsten betroffen. Speziell in Hamburg beträgt der prozentuale Anteil der unter 59-Jährigen bis zu diesem Zeitpunkt 78,7 % von insgesamt 47.191 infizierten Personen [28]. Gemäß Berichten des RKI sind bis zum 29.12.2020^2^ in Deutschland insgesamt 30.978 Personen an COVID-19 verstorben, wobei der Anteil der über 70-Jährigen 88 % beträgt (Median 84 Jahre). 322 der Verstorbenen (1,04 %) waren zwischen 0 und 49 Jahre alt (112 weiblich, 210 männlich). Bis zu diesem Datum waren dem RKI 13 Todesfälle von unter 20-Jährigen gemeldet worden [29].

Internationale Studien sowie die eigene Auswertung zeigen ähnliche Ergebnisse auf. Guan et al. untersuchten Daten von 1099 Patienten, welche mit SARS-CoV‑2 infiziert waren, mit einem Durchschnittsalter von 47 Jahren in China. 566 der Personen waren unter 50 Jahre alt. 15 (1,4 %) Personen verstarben; konkrete Altersangaben zu diesen Sterbefällen liegen nicht vor [5]. In einer italienischen multizentrischen Beobachtungsstudie wurden bei 3894 Patienten 712 SARS-CoV-2-assoziierte Todesfälle dokumentiert, von denen 6 Personen in einem Alter zwischen 18 und 44 Jahren waren. 69 der 712 Verstorbenen waren zudem übergewichtig [3]. Ioannidis et al. untersuchten bis Ende April 2020 in 14 Ländern und 13 US-Bundesstaaten SARS-CoV-2-assoziierte Todesfälle und verglichen in ihrer Studie Personen unter 65 Jahren mit Personen über 65 Jahren. In den europäischen Ländern und in Kanada machten die unter 65-jährigen Verstorbenen einen Anteil von 4,5–11,2 %, in den US-Bundesstaaten einen Anteil von 8,3–22,7 % aller COVID-19-Todesfälle aus. Personen in einem Alter unter 40 Jahren waren in weniger als 1,3 % in den europäischen Ländern und Kanada sowie in 0,4–2,3 % in den US-Bundesstaaten verstorben. In den europäischen Ländern und Kanada hatten Menschen unter 65 Jahren insgesamt ein 30- bis 100-fach geringeres Risiko, an COVID-19 zu sterben, als Menschen über 65 Jahren; für die US-Standorte war die relative Sterblichkeitsrate etwas geringer (16- bis 52-fach) [8].

Neben höherem Lebensalter als Risikofaktor für schwere Pneumonien haben auch Komorbiditäten [9, 18, 22] einen Einfluss auf die Hospitalisierungsrate und die Letalität bei SARS-CoV‑2. Hier zeigten sich insbesondere arterielle Hypertonie, Diabetes mellitus und die koronare Herzerkrankung als Einflussfaktoren für eine erhöhte Letalität [14, 26]. Ein weiterer Risikofaktor für schwere COVID-19-Erkrankungsverläufe scheint, im Ergebnis der Datenlage, insbesondere bei jüngeren Personen, die morbide Adipositas zu sein. Bekannt ist, dass Adipositas generell mit einer erhöhten Sterblichkeitswahrscheinlichkeit bei infektiösen Erkrankungen, auch unabhängig von SARS-CoV‑2, assoziiert ist [30–32]. Übergewicht ist eine der wichtigsten chronischen Erkrankungen [33] und gilt als unabhängiger Risikofaktor für die Schwere einer SARS-CoV-2-Infektion, zumal Personen mit Übergewicht in fortgeschrittenem Alter regelhaft kardiovaskuläre Komorbiditäten aufweisen [18, 34]. Das Fettgewebe selbst kommt als Reservoir für SARS-CoV‑2 und als möglicher Ort einer Virusreplikation in Betracht. Eigene hierzu laufende Laboranalysen müssten dann über Kohortenstudien mögliche statistisch relevante Effekte in COVID-19-Patienten herausarbeiten. Der Risikofaktor Übergewicht für eine schwere Erkrankung bis hin zum tödlichen Verlauf insbesondere bei jungen Personen konnte jedoch bereits in einigen Studien nachgewiesen werden [18, 30–32].

In der Untersuchung von Rao et al. waren 114 von 240 an SARS-CoV‑2 erkrankten Patienten übergewichtig. In dieser Studie korrelierten höhere BMI-Werte mit schweren Krankheitsverläufen. Im Gegensatz zu den normalgewichtigen Erkrankten litten die übergewichtigen Personen vermehrt an Dyspnoe, Brustschmerz und entwickelten schneller Pneumonien. Letztendlich konnte jedoch kein signifikanter Unterschied zwischen dem Outcome (Entlassung oder Versterben) bei über- oder normalgewichtigen Patienten festgestellt werden. Bezüglich des Auftretens der Krankheitssymptome und des Ansprechens auf eine medizinische Behandlung existieren jedoch signifikante Unterschiede zwischen normal- und übergewichtigen Patienten. Lediglich 2 der insgesamt 25 verstorbenen Personen waren unter 45 Jahre alt, wobei eine Person über- und eine normalgewichtig war [18].

Die hier präsentierte Studie zeigt, dass alle der in Hamburg untersuchten, *mit* oder *an *SARS-CoV‑2 verstorbenen Personen bis 50 Jahre schwer vorerkrankt waren. In 10 der 12 Todesfälle führte letztendlich die Infektion mit SARS-CoV‑2 zum Versterben. Personen im jüngeren Erwachsenenalter ohne relevante kardiovaskuläre Vorerkrankungen oder andere Komorbiditäten sind in den Hamburger Kollektiven aus dem Jahr 2020 nicht dokumentiert.

Die Hauptlimitation der Untersuchung besteht in der kleinen Fallzahl von 3 Verstorbenen mit Hamburger Meldeadresse und 3 mit externer Meldeadresse aus dem vergleichsweise großen Sterbefallkollektiv in Hamburg (bezogen auf die Sterbefälle mit Hamburger Meldeadresse: 1,2 % (9/735) aller SARS-CoV-2-assoziierten Todesfälle bzw. 1,3 % (8/618) aller COVID-19-Todesfälle). Zu vergleichbaren Ergebnissen mit einem Anteil der unter 44-Jährigen von 0,8 % aller Todesfälle waren Di Castelnuovo et al. gekommen [3]. Eine statistisch validierte Evaluation von prädiktiven Risikofaktoren für das Versterben an COVID-19 bei jungen Personen ist erst bei einer größeren Fallzahl oder perspektivisch durch Metaanalysen möglich.

Die Untersuchung der SARS-CoV-2-assoziierten Todesfälle in Hamburg und die international veröffentlichten Untersuchungen [2, 4, 6, 22, 35] machen deutlich, wie wichtig rechtsmedizinische und pathologische Obduktionen der SARS-CoV‑2 Todesfälle sind, um neue Erkenntnisse über die Krankheitsverläufe und Todesursachen zu gewinnen und damit einen wissenschaftlichen Beitrag für die Pandemiebekämpfung zu leisten.

## Schlussfolgerung

- Die *mit* oder *an *SARS-CoV‑2 verstorbenen Personen bis 50 Jahre in Hamburg waren schwer vorerkrankt.
- 1,3 % der Hamburger COVID-19-Todesfälle betreffen Personen bis 50 Jahre. Vier der 12 Verstorbenen waren nach WHO-Kriterien adipös (Adipositas, Grade I und III); weitere 4 waren präadipös.
- In der Hälfte der COVID-19-Sterbefälle wurden autoptisch thrombembolische Ereignisse festgestellt.
- Auch postmortal sind (ggf. sogar verdachtsunabhängige) PCR-Screenings auf SARS-CoV‑2 zum Infektionsnachweis sinnvoll.

## Footnotes

1 Ursächlich für die obere gastrointestinale Blutung war ein durch die ketoazidotische Stoffwechsellage ausgelöstes Erbrechen bei Diabetes mellitus Typ 1.

2 In den Situationsberichten des Robert Koch-Instituts sind lediglich bis zum 29.12.2020 die Altersklassen der Sterbefälle verfügbar.
